# Autophagy inhibition and reactive oxygen species elimination by acetyl-CoA acetyltransferase 1 through fused in sarcoma protein to promote prostate cancer

**DOI:** 10.1186/s12885-022-10426-5

**Published:** 2022-12-14

**Authors:** Jingqian Guan, Xizi Jiang, Yaoxing Guo, Wenhui Zhao, Ji Li, Yizhuo Li, Ming Cheng, Lin Fu, Yue Zhao, Qingchang Li

**Affiliations:** 1 Department of Pathology, College of Basic Medical Sciences, China Medical University, Shenyang, Liaoning Province People’s Republic of China; 2 Department of Pathology, The First Hospital of China Medical University, No. 155 NanjingBei Street, Heping District, Shenyang, Liaoning Province People’s Republic of China

**Keywords:** Autophagy, Reactive oxygen species, ACAT1, FUS, Prostate cancer

## Abstract

**Background:**

Prostate cancer is a major health issue affecting the male population worldwide, and its etiology remains relatively unknown. As presented on the Gene Expression Profiling Interactive Analysis database, acetyl-CoA acetyltransferase 1 (ACAT1) acts as a prostate cancer-promoting factor. ACAT1 expression in prostate cancer tissues is considerably higher than that in normal tissues, leading to a poor prognosis in patients with prostate cancer. Here, we aimed to study the role of the ACAT1-fused in sarcoma (FUS) complex in prostate cancer and identify new targets for the diagnosis and treatment of the disease.

**Methods:**

We conducted immunohistochemical analysis of 57 clinical samples and in vitro and in vivo experiments using a mouse model and plasmid constructs to determine the expression of ACAT1 in prostate cancer.

**Results:**

The relationship between the expression of ACAT1 and the Gleason score was significant. The expression of ACAT1 was higher in tissues with a Gleason score of > 7 than in tissues with a Gleason score of ≤7 (*P =* 0.0011). In addition, we revealed that ACAT1 can interact with the FUS protein.

**Conclusions:**

In prostate cancer, ACAT1 promotes the expression of P62 and Nrf2 through FUS and affects reactive oxygen species scavenging. These effects are due to the inhibition of autophagy by ACAT1. That is, ACAT1 promotes prostate cancer by inhibiting autophagy and eliminating active oxygen species. The expression of ACAT1 is related to prostate cancer. Studying the underlying mechanism may provide a new perspective on the treatment of prostate cancer.

**Supplementary Information:**

The online version contains supplementary material available at 10.1186/s12885-022-10426-5.

## Background

Prostate cancer is the second most frequently diagnosed malignancy and was the fifth leading cause of cancer-related deaths in men worldwide in 2020 [1, 2]. An increasing number of young men have been suffering from prostate cancer [3]. Therefore, the prevention and treatment of prostate cancer are essential. Commonly used treatment methods for prostate cancer include surgical treatment, non-invasive treatment, and androgen therapy [4–6]. With emerging treatment methods, such as immunotherapy and molecular therapy [7–9], the treatment of prostate cancer and the prognosis have improved. However, prostate cancer prevention and early treatment options are still research hotspots [10].

Acetyl-CoA acetyltransferase 1 (ACAT1) is an important enzyme [11–17] as it plays a vital role in various cancers, for example, by exerting a liver [18] and kidney tumor-promoting effect and an anti-ovarian cancer effect [19, 20]. It also plays a prostate cancer-promoting role [21]; however, the detailed mechanism has not yet been elucidated.

The role of the fused in sarcoma (FUS) RNA-binding protein, responsible for the regulation of RNA, has been reported in amyotrophic lateral sclerosis (ALS) and frontotemporal dementia [22–24], but there are only a few reports on its role in cancer. FUS is reportedly related to prostate cancer, which inhibits tumor proliferation [25]. Therefore, in prostate cancer, FUS is regarded as a tumor suppressor. Data from the STRING database show that ACAT1 can interact with FUS; hence, the underlying mechanism of the interaction requires an in-depth discussion. Here, we aimed to study the role of the ACAT1-FUS complex in prostate cancer and identify new targets for the diagnosis and treatment of the disease.

## Methods

### Patients and specimens

A total of 57 prostate cancer clinical tissue samples were used in this study; they were obtained from the First Affiliated Hospital of China Medical University. The Gleason score was determined at the Department of Pathology, the First Affiliated Hospital of China Medical University, and the samples were collected between 2013 and 2017. This study was approved by the Ethics Committee of China Medical University, and all patients signed informed consent forms.

### In vivo nude mouse experiments

The experimental protocol for the animal experiments in this study was approved by the Institutional Animal Care and Use Committee of China Medical University (CMU2021546) and adhered to the guidelines for the care and use of laboratory animals issued by the China Animal Research Council. Four-week-old female *BALB/c* nude mice were purchased from Charles River and housed in specific pathogen-free (SPF) “barrier” facilities. We injected 1 × 10^7^ PC3 cells stably overexpressing ACAT1 under the right creaking fossa of each female mouse. The mice were raised for 1 month. During this period, SPF mice were provided chow and allowed to drink sterile water ad libitum. The temperature was maintained at 22–26 °C and suitable humidity was maintained under a 12−/12-h light/dark cycle. After the end of the experiment (1 month), the tumor was excised from each mouse, the weight and size of the tumors were measured, and statistical analysis was performed.

### Cell culture

All cell lines were obtained from the Shanghai Cell Bank (Shanghai, China) and cultured in RPMI 1640 medium with fetal bovine serum (FBS; FB15015; Clark Biosciences, Richmond, VA, USA). LNCaP and PC3 cells were cultured in a medium containing 10% FBS and no antibiotics, according to the manufacturer’s instructions, and maintained in a 5% CO_2_ incubator at 37 °C.

### Plasmid construction and transfection

ACAT1 siRNA (siB170726094953-1-5) and normal control cells were purchased from Ruibo Biosciences (Guangzhou, China). The overexpression plasmid ACAT1-3Flag of ACAT1 and its control GV141 were obtained from GENE (Shanghai, China). MAP 1LC3B-Flag (RC207356) was obtained from Origene (Beijing, China). The cells were transfected using Lipofectamine 3000 reagent (Invitrogen, Carlsbad, CA, USA) according to the manufacturer’s instructions.

### Immunohistochemistry and Gleason scores

The 57 tissue sections were incubated with ACAT1 rabbit polyclonal antibody (16215-AP-1, 1:100; ProteinTech Group, Rosemont, IL, USA) at 4 °C overnight. Thereafter, they were incubated with the secondary antibody for 2 h at 37 °C. The nuclei were stained with hematoxylin for 10 min; 100 μL of 3,3′-diaminobenzidine chromogenic solution (P0202, Beyotime Biotechnology, Shanghai, China) was added to each tissue section and ACAT1 expression was observed under a microscope. Next, the tissue sections were strictly scored according to the latest Gleason scoring system. The χ^2^ test was used to determine the correlation between ACAT1 expression and clinicopathological characteristics. *P <* 0.05 was considered to indicate a statistically significant difference.

### Western blotting

The total cell protein was extracted using lysis buffer (P0013; Beyotime Biosciences, Shanghai, China). We added a mixture of protease and phosphatase inhibitors (B14002 and B15002; Biotool, Shanghai, China), four times the volume of the cell pellet, to the cell lysate. The total protein (35 μg) was separated via SDS-PAGE and transferred onto a polyvinylidene fluoride membrane (EMD-Millipore, Billerica, MA, USA). The membrane was blocked with 5% skimmed milk (232,100; BD Biosciences, Franklin Lakes, NJ, USA) for at least 2 h and then incubated with the appropriate primary antibody overnight at 4 °C. Peroxidase-conjugated secondary antibody was added and incubated at 37 °C for 1 h. The following primary antibodies were used: Anti-ACAT1 (16215-AP-1, 1:1000), anti-P62/SQSTM1 (66184-1-lg, 1:1000), anti-NRF2 (16396-1-AP, 1:1000), anti-lamin B1 (66095-1-lg, 1:1000), anti-FUS (60160-1-lg, 1:1000), and anti-GAPDH (60004-1-Ig, 1:1000) from ProteinTech Group, and anti-LC3B (3868 s, 1:1000) from Cell Signaling Technology (Denver, MA, USA). The total protein was visualized using an enhanced chemiluminescence method (34,080; Thermo Fisher Scientific, Waltham, MA, USA). Image J software (National Institutes of Health, Bethesda, MD, USA) was used to evaluate each band quantitatively and Prism 5.0 software (GraphPad, La Jolla, CA, USA) was used to compare the signal intensity.

### Quantitative real-time polymerase chain reaction (q-PCR)

q-PCR was performed using SYBR Green PCR Master Mix in a 7900HT Fast Real-Time PCR System (Applied Biosystems, Foster City, CA, USA) with a total reaction volume of 20 μL. The q-PCR conditions were as follows: 95 °C for 30 s, 95 °C for 5 s, and 60 °C for 30 s, for 40 cycles. The dissociation step was used to generate a melting curve and confirm amplification specificity. The expression level relative to β-actin expression was calculated using the 2-ΔΔCt method.

### Co-immunoprecipitation assays

The cell lines used in the experiment were seeded in two 10-cm cell culture dishes. When the cells reached confluence, they were lysed for 20 min and centrifuged at 12000 rpm for 15 min at 4 °C. Next, 40 μL of protein A/G Sepharose (P2012; Beyotime Biosciences) was added to the supernatant and blocked for at least 2 h. The mixture was then centrifuged at 1000 rpm for 5 min at 4 °C, and the supernatant was divided into two parts. Anti-ACAT1 or anti-FUS antibody (8 μg) was added to one part, and anti-mouse/rabbit IgG (1:2000; ZSGB-BIO, Beijing, China) to the other part. The mixture was shaken overnight at 4 °C. The next day, 25 μL of agarose A/G magnetic beads was added to each tube and incubated at 4 °C for 6 h. The cell lysate was then washed, heated in boiling water for 10 min, and finally, immunoblotting was performed.

### Immunofluorescence

Immunofluorescence co-localization experiments were performed with PC3 and LNCaP cell lines. The two prostate cancer cell lines were fixed in a glass-bottomed dish with paraformaldehyde, and then anti-ACAT1 (1:50) and anti-FUS (1:50) antibodies were added and incubated for 16 h. The next day, the secondary antibody was added and incubated for 2 h at 37 °C, the nucleus was stained with DAPI, and finally, the image was acquired using a laser confocal microscope (FV3000, Olympus, Tokyo, Japan).

### Nucleoplasmic separation

The target cells were collected and separated from the cytoplasm and nucleoproteins. The experiment was then performed according to the instructions provided by the manufacturer, using a nuclear and cytoplasmic protein extraction kit (P0027, Beyotime Biosciences). The separated proteins were then boiled for 5 min and subjected to western blotting.

### EdU cell proliferation assays

LNCap or PC3 cells stably transfected with FUS or ACAT1 were added to a 35-mm cell culture dish, and after overnight culture, the corresponding reagents were added according to the instructions of Edu (C0075L; Beyotime Biotechnology, Shanghai, China), and finally photographed using a confocal laser microscope and statistical analysis was performed.

### Transwell cell migration experiment

Stably transfected cell lines were seeded (5 × 10^4^ cells) into Transwell chambers (Costar, Washington, DC, USA). The Transwell chambers were plated with Matrigel (Corning Life Sciences, Corning, NY, USA), 1 day in advance, and then the Transwell chambers with 5 × 10^4^ cells were placed in a 24-well culture plate with 600 μL of FBS for 24 h. After 24 h, the Transwell chambers were washed thrice with pre-cooled PBS, the cells were fixed with pre-cooled methanol for 10 min, and then washed an additional three times with PBS. After washing, the cells were stained with crystal violet for 10 min. Micrographs were captured using CellScan software, and the cells were counted using PS software. Finally, GraphPad Prism 5 was used to analyze the obtained data. Statistical significance was set at *P* < 0.05. This experiment was performed in triplicates.

### Active oxygen detection assays

The level of reactive oxygen species (ROS) generated by prostate cancer cells was detected using the probe 2′,7′-dichlorodihydrofluorescein (DCFH-DA, Beyotime Biotechnology), which detected diacetate. After 48 h of transfection with the SIRT5 plasmid or siRNA, the cells were cultured in the dark for 20 min with 10 μM DCFH-DA in a humidified atmosphere at 37 °C in the presence of 5% CO_2_. The cells were washed three times with cold phosphate-buffered saline to remove excess fluorescent probes. The cells were counted, 10^4^ cells were added to each well of a 96-well plate, and the absorbance was measured at 488 nm.

### Chromatin immunoprecipitation (ChIP)

For ChIP, 2 × 10^7^ PC3 and LNCaP cells overexpressing LC3B were used. The experiment was performed using the Millipore ChIP kit (17-371, Boston, MA, USA) according to the manufacturer’s instructions. Finally, micrographs were obtained using Image Lab software. The antibody used was anti-Flag (66008-4-lg, ProteinTech). The primers used were FUS forward, 5′-CGTCCCCAGCCGCCGGGACCG-3′ and reverse, 5′-ACGCTCCGCCGCCTACGCACCG-3′.

### Databases

In this study, we used Gene Expression Profiling Interactive Analysis (GEPIA) (http://gepia.cancer-pku.cn/) and STRING: functional protein association networks (https://string-db.org/). The GEPIA database was used to analyze ACAT1 expression in and prognosis of prostate cancer, and the STRING database was used to analyze the interaction between proteins.

### Statistical analysis

All data were analyzed using SPSS version 24.0 (Beijing, China) to perform the χ^2^ tests, and Prism 5 (GraphPad) software was used for ROS analysis and signal intensity analysis in western blotting experiments. All experiments were repeated at least three times independently under the same conditions. Results with *P <* 0.05 were considered statistically significant.

## Results

### High ACAT1 expression in prostate cancer cells correlates with the tumor Gleason score

**Fig. 1:**
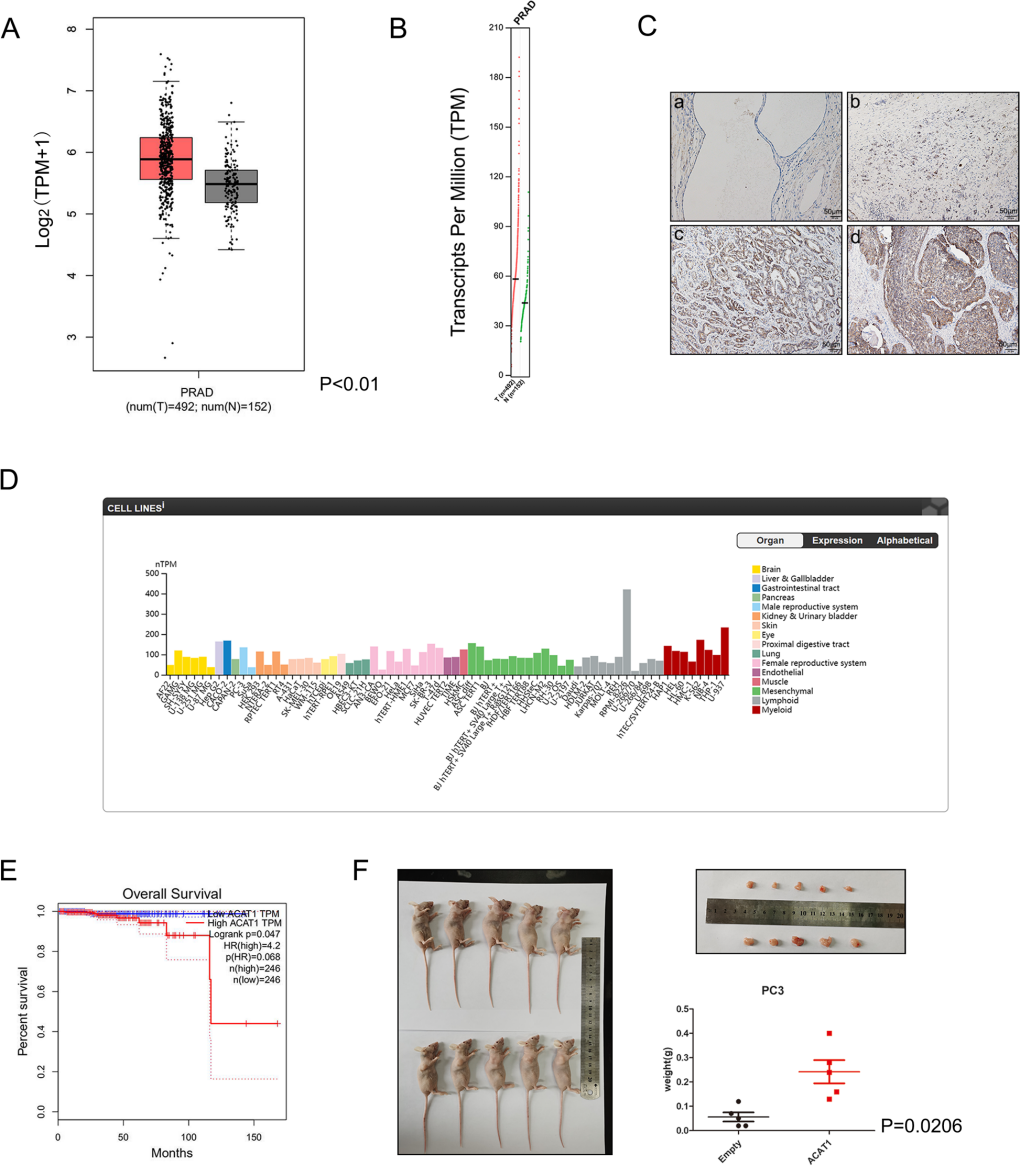
High expression of ACAT1 in prostate cancer cells correlates with the tumor Gleason score. **A** and **B**. From the GEPIA database, the results show that in prostate adenocarcinoma (PRAD), the expression of ACAT1 is significantly higher than that in the adjacent normal tissues (T on the left, N on the right). **C**. Expression of ACAT1 in prostate tissue sections. (a) Normal prostate gland. (b) Prostate cancer tissue with a Gleason score of 3. (c) Prostate cancer tissue with a Gleason score of 4. (d) Prostate cancer tissue with a Gleason score of 5. **D**. From the Human Protein Atlas, display of ACAT1 expression in prostate cancer cell lines. **E**. From the GEPIA database, the results show that in prostate cancer, high expression of ACAT1 is associated with a poor prognosis. **F**. Tumorigenic ability of PC3 cell line with stable and high expression of ACAT1 in nude mice compared with that in the negative control group (*P =* 0.0206)

Through the GEPIA database, we found that *ACAT1* is an oncogene in prostate cancer (Fig. 1A and B). Therefore, we conducted a series of experiments to elucidate the prostate cancer-promoting role of *ACAT1*.

First, we randomly selected 57 clinical samples for the immunohistochemistry experiments. In these clinical samples, we found that ACAT1 expression was higher in prostate cancer tissues than in normal prostate tissues (Fig. 1C), and this was in line with the data obtained from the GEPIA. The data from the database and ACAT1 expression in prostate cancer were correlated with the Gleason score (Table 1). The results showed that when the Gleason score was 7, irrespective of whether it was 3 + 4 or 4 + 3, there was no significant difference in ACAT1 expression (*P =* 0.7968). When the Gleason scores were 6 and 7, there was no significant difference in ACAT1 expression (*P =* 0.3126). Similarly, the differences in ACAT1 expression between the groups with Gleason scores of 8 and 9 were also not significant (*P =* 0.6500). Only between groups with Gleason scores greater than 7 and those with Gleason scores of 7 or less, the difference was significant (*P =* 0.0011). Through The Human Protein Atlas database, we found that the expression of ACAT1 was highly objective in PC3 cells, which represent cells of the male reproductive system (Fig. 1D). Therefore, we chose the PC3 cell line for the experiment. In the following experiments, LNCaP, as a hormone-dependent prostate cancer cell line, and PC3, as a hormone-independent cell line, were used to represent prostate cancer. At the same time, through the GEPIA database, we found that high ACAT1 expression was associated with a poor prognosis in patients with prostate cancer (Fig. 1E, *P =* 0.047). Through tumorigenic experiments in nude mice, we learned that the PC3 cell line with high expression of ACAT1 had tumorigenic properties, and the tumor weight was high (Fig. 1F, *P =* 0.0206). With increased ACAT1 expression, the proliferation (Fig. S1A) and migration (Fig. S1B) of prostate cancer cell lines enhanced. These results indicate that ACAT1 plays a prostate cancer-promoting role.

**Table 1:** Expression of ACAT1 in prostate cancer tissues

	Number	ACAT1 overexpression	ACAT1 negative or weak expression	***p-***value
Age				
≤ 65	30	19	11	*P =* 0.5501
> 65	27	15	12	
Gleason score				
≤ 7	35	15	20	*P =* 0.0011
> 7	22	19	3	
6	22	8	14	*P =* 0.3126
7	13	7	6	
8	12	10	2	*P =* 0.6500
9	10	9	1	
3 + 4	6	3	3	*P =* 0.7968
4 + 3	7	4	3	
6	22	8	14	*P =* 0.0007
8 and 9	22	19	3	
7	13	7	6	*P =* 0.0334
8 and 9	22	19	3	

### ACAT1 can bind to FUS

**Fig. 2:**
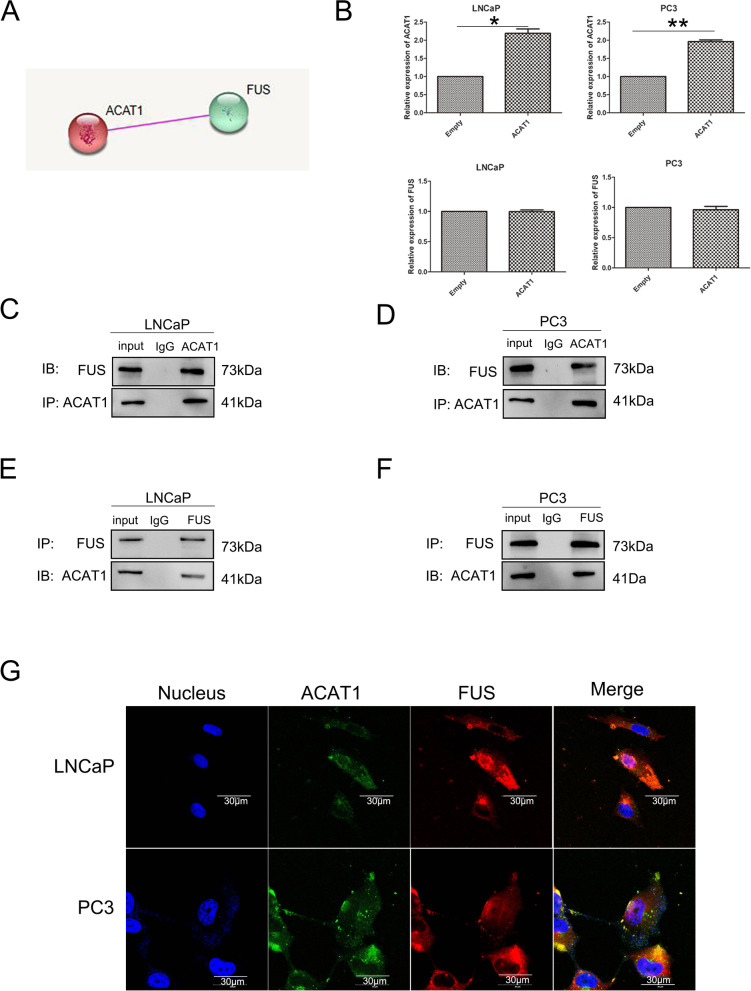
ACAT1 can bind to FUS. **A**. From the STRING database, we found that ACAT1 can interact with FUS. **B**. Changes in the *FUS* mRNA level after an increase in ACAT1 expression, as determined using q-PCR. *P < 0.05, **P < 0.01. **C** and **D**. In LNCaP and PC3 cells, the endogenous ACAT1 can bind to FUS. **E** and **F**. In LNCaP and PC3 cells, the endogenous FUS can interact with ACAT1. **G**. Immunofluorescence experiments showing co-localization of ACAT1 and FUS in PC3 and LNCaP cell lines

To elucidate the prostate cancer-promoting role of ACAT1, we screened the STRING database. We found that ACAT1 interacted with the FUS protein (Fig. 2A).

To elucidate how ACAT1 interacts with FUS, we conducted q-PCR analysis and found that FUS mRNA expression did not change significantly after ACAT1 upregulation in both LNCaP and PC3 cell lines (Fig. 2B). This indicates that the regulation of ACAT1 on FUS did not occur at the transcriptional level; therefore, we investigated the correlation of their expression at the protein level. Through western blotting, we found that there was negative correlation between the expression of ACAT1 and that of FUS in four common prostate cancer cell lines (LNCaP, PC3, DU145, and 22RV1) (Fig. S2A，*R* = -0.9148). Through co-immunoprecipitation, we found that endogenous ACAT1 could interact with FUS in either LNCaP or PC3 cells (Fig. 2C and D). At the same time, in these two cell lines, the endogenous FUS could bind to ACAT1 (Fig. 2E and F). Through laser confocal microscopy, we observed that FUS could co-localize with ACAT1 in the cytoplasm in prostate cancer cells, and FUS not bound to ACAT1 existed in the nucleus of some prostate cancer cells (Fig. 2G).

### ACAT1 scavenges ROS in prostate cancer

**Fig. 3:**
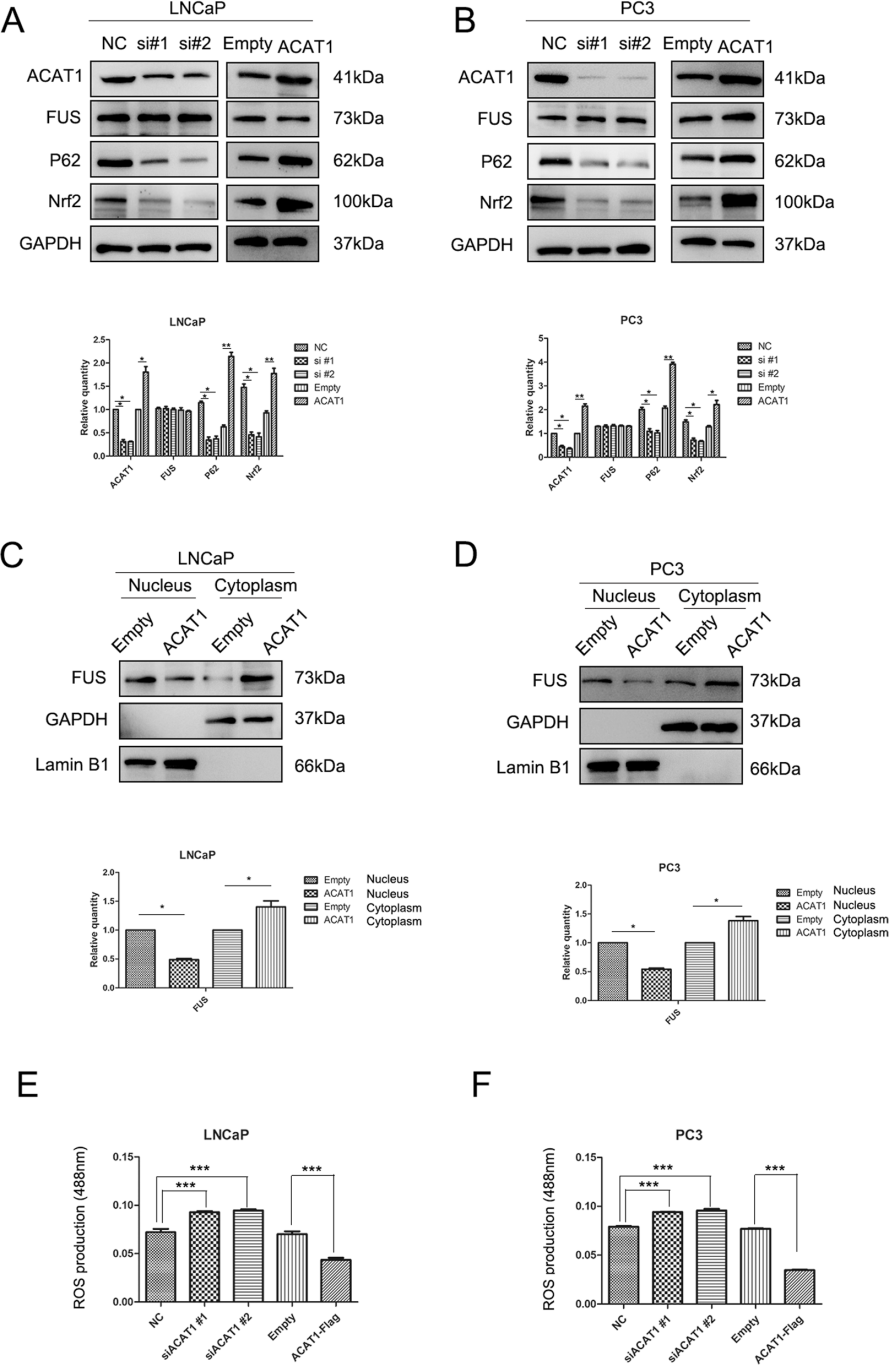
ACAT1 scavenges ROS in prostate cancer. **A** and **B**. ACAT1 modulates the expression of functional and pathway proteins in (A) LNCaP and (B) PC3 cells. Si#1 and si#2 represent siRNA 1 and siRNA 2, respectively. The gray value statistics chart is shown. *P < 0.05, **P < 0.01. **C** and **D**. In LNCaP (C) and PC3 (D) cells, FUS enters the nucleus. The statistical chart of gray values is shown. *P < 0.05, **P < 0.01. **E** and **F**. Highly expressed ACAT1 scavenges ROS in LNCaP (E) and PC3 (F) cells. *P < 0.05, **P < 0.01

In previous studies, we found that sirtuin 5 (SIRT5) can regulate the expression of ROS [26], and ACAT1 can bind to and be regulated by SIRT5 [21]. We sought to clarify the role of ACAT1 in prostate cancer by regulating ACAT1 expression in LNCaP and PC3 cell lines. We found that when ACAT1 expression decreased, Nrf2 and P62 expression also decreased. Similarly, when ACAT1 expression increased, the expression of the two proteins also increased (Fig. 3A and B); however, FUS expression did not change notably when ACAT1 expression was upregulated or inhibited. However, after conducting nucleoplasmic separation experiments, we found that when ACAT1 expression increased, FUS expression in the nucleus decreased. This result was verified in both LNCaP and PC3 cell lines (Fig. 3C and D). At the same time, ACAT1 was always present in the cytoplasm of tumor cells (Fig. S2B). Although ACAT1 did not regulate the changes in total FUS expression in the cells, after the binding of ACAT1 to FUS, it prevented FUS from entering the nucleus. By analyzing ROS, we found that ACAT1 acted as an ROS scavenger (Fig. 3E and F). This might have led to its prostate cancer-promoting effect.

### ACAT1 inhibits autophagy of prostate cancer through FUS and exerts a tumor-promoting effect

**Fig. 4:**
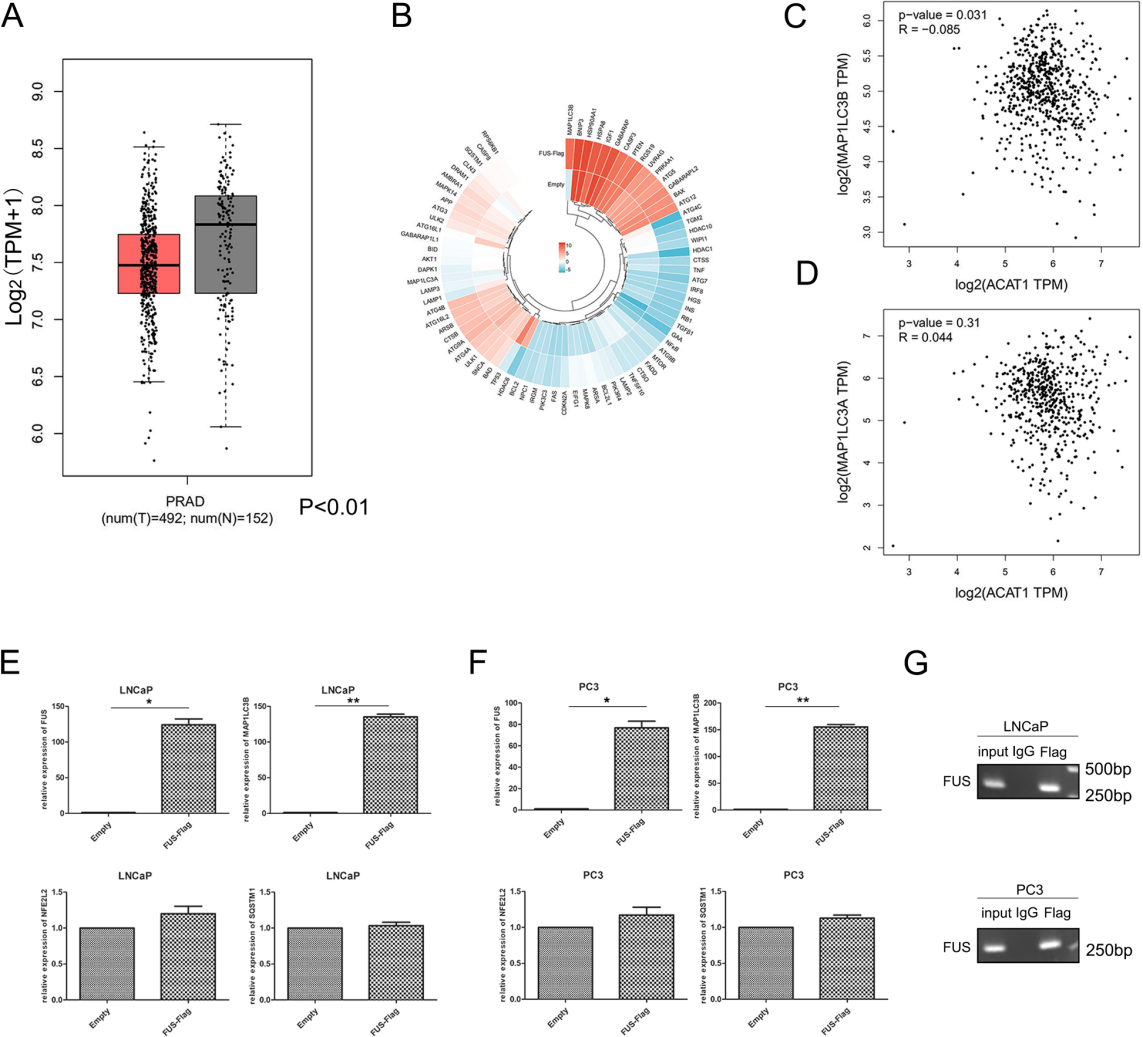
ACAT1 inhibits the autophagy of prostate cancer cells through FUS. **A**. Expression of FUS in the GEPIA database. **B**. Autophagy-related qPCR array. After upregulation of the expression of FUS, the changes in the expression of related genes are shown. **C** and **D**. Relationship between ACAT1 and *MAP 1LC3B* (*P =* 0.031, *R* = − 0.085), as well as ACAT1 and *MAP 1LC3A* (*P =* 0.31, *R* = 0.044), in the GEPIA database. **E**. In LNCaP cell line, when *FUS* expression increases, *MAP 1LC3B* mRNA expression increases accordingly. *P < 0.05, **P < 0.01. **F**. In PC3 cell line, when *FUS* expression increases, *MAP 1LC3B* mRNA expression increases. *P < 0.05, **P < 0.01. **G**. ChIP experiments demonstrate that FUS can interact with LC3B

From the GEPIA database, we found that the average expression of FUS in prostate cancer was lower than that in the adjacent normal prostate tissues (Fig. 4A). To verify whether FUS can regulate the autophagy of prostate cancer, we used an autophagy PCR array. Among many genes related to autophagy, we identified *LC3B*, with the most obvious expression change after the upregulation of FUS (Fig. 4B). From the GEPIA database, we also learned that ACAT1 expression is negatively correlated with LC3B expression (Fig. 4C), but the relationship of ACAT1 expression with LC3A expression was not significant (Fig. 4D). Although the degree of negative correlation between ACAT1 and LC3B expression in the database was small (*R* = − 0.085), it was significant (*P =* 0.031), and such results would still be valuable. We also verified whether ACAT1 and LC3B expression is affected by FUS. We conducted q-PCR and found that, in LNCaP and PC3 cell lines, when FUS expression increased, the mRNA expression of LC3B (*MAP 1LC3B*) increased significantly, but that of P62 (*SQSTM1*) and Nrf2 (*NFE2L2*) did not change significantly (Fig. 4E and F). We also performed ChIP experiments and found that FUS could bind to LC3B (Fig. 4G). In addition, another unanswered question is whether the expression of ACAT1 was increased alone (Fig. S2C) or the expression of ACAT1 and FUS was increased simultaneously (Fig. S2D), and we found that the expression of *NFE2L2* and SQSTM1 did not change significantly.

**Fig. 5:**
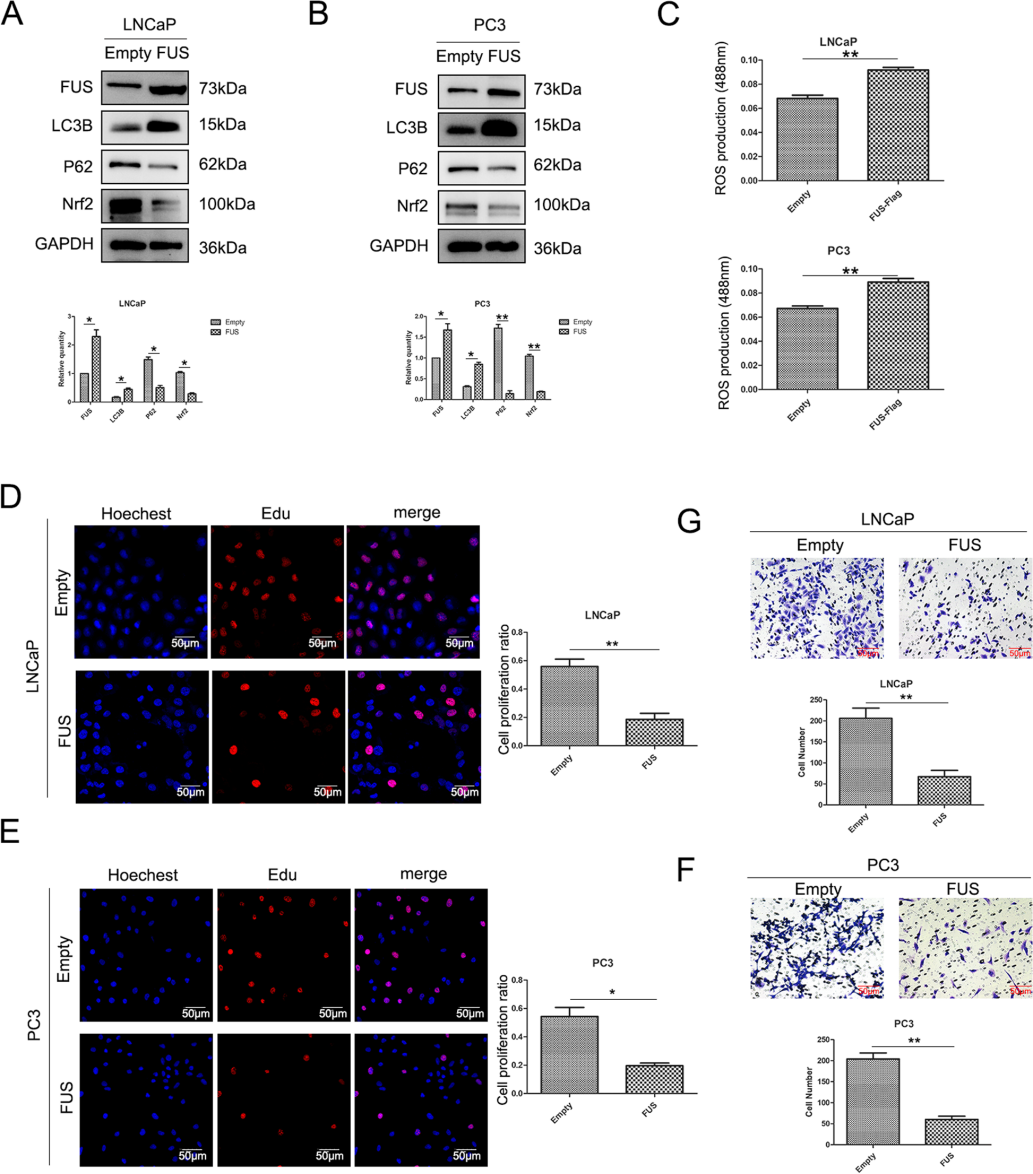
FUS is a suppressor of prostate cancer. **A** and **B**. Western blotting showing high expression of FUS in LNCaP (A) and PC3 (B) cells, which promotes autophagy. *P < 0.05, **P < 0.01. **C**. FUS causes the accumulation of ROS in LNCaP and PC3 cells; LNCaP at the top, and PC3 at the bottom. *P < 0.05, **P < 0.01. **D** and **E**. Edu assay, the change of (D) LNCaP and (E) PC3 cells proliferation ability after FUS expression increased.*P < 0.05, **P < 0.01. **F** and **G**. Transwell cell migration assay, changes in the migration ability of (F) LNCaP cells and (G)PC3 cells after the expression of FUS increased.*P < 0.05, **P < 0.01

With the increase in FUS expression, the expression of Nrf2 and P62 decreased, whereas that of LC3B increased; furthermore, Nrf2 and P62 expression was significantly decreased (Fig. 5A and B). When the expression of ACAT1 alone increased, the expression of LC3B decreased (Fig. S2E), and ROS accumulation increased (Fig. 5C). When the expression of FUS increased, the proliferative capacity of the cells decreased (Fig. 5D and E), which was related to the accumulation of ROS induced by FUS. FUS also reduced the migration of LNCaP and PC3 cells (Fig. 5F and G). Combined with the above results, we found that FUS promoted autophagy by regulating LC3B expression, and inhibited the proliferation and migration of prostate cancer.

**Fig. 6:**
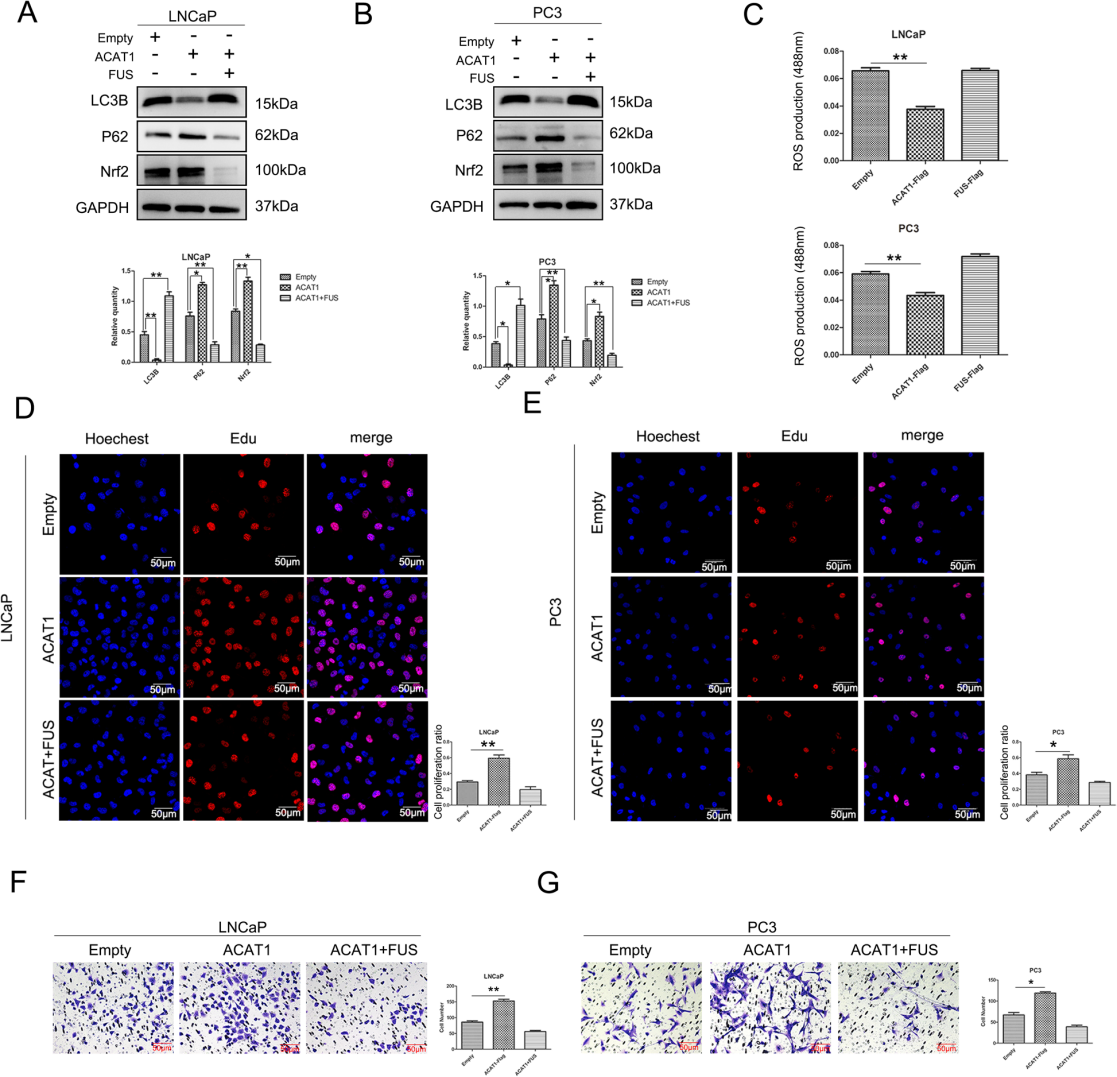
ACAT1 plays a tumor-promoting role through FUS. **A** and **B**. After a simultaneous increase in the expression of ACAT1 and FUS, the expression of the related proteins changed in (A) LNCaP and (B) PC3 cells. The statistical analysis of gray values is shown. *P < 0.05, **P < 0.01. **C** After a simultaneous increase in the expression of ACAT1 and FUS, the accumulation of ROS is shown. LNCaP on the top and PC3 on the bottom. *P < 0.05, **P < 0.01. **D** and **E**. Edu assay, after a simultaneous increase in the expression of ACAT1 and FUS,the change of (D) LNCaP and (E) PC3 cells proliferation ability *P < 0.05, **P < 0.01. **F** and **G**. Transwell cell migration assay, after a simultaneous increase in the expression of ACAT1 and FUS,changes in the migration ability of (F) LNCaP cells and (G)PC3 cells .*P < 0.05, **P < 0.01

Nrf2 and P62 expression was significantly decreased and LC3B expression was upregulated following simultaneous overexpression of ACAT1 and FUS in LNCaP and PC3 cell lines compared with that when ACAT1 alone was overexpressed (Fig. 6A, B, S3A, and S3B). This indicated that FUS inhibited the effect of ACAT1. We determined the level of ROS and found that the accumulation of ROS increased (Fig. 6C). This finding indicated that the regulatory effect of ACAT1 on the accumulation of ROS was also achieved through FUS. In addition, the proliferation (Fig. 6D and E) and migration (Fig. 6F and G) of the cells were significantly reduced. Therefore, we believe that ACAT1 plays a tumor-promoting role by preventing FUS from transcribing *LC3B*.

## Discussion

Here, we showed that ACAT1 expression was higher in prostate cancer tissues, especially in high-grade tumors with Gleason scores of 8 and 9 than in normal tissues. Moreover, high ACAT1 expression was related to the poor prognosis of patients with prostate cancer. This finding shows that ACAT1 has an important role in the development of prostate cancer. As younger individuals are developing prostate cancer, its prevention, early detection, and treatment are vital [3].

In this study, we found that ACAT1 inhibited autophagy in prostate cells and exerted an ROS-scavenging effect. Both autophagy and increased ROS levels have certain tumor-inhibiting effects [27–29], and ACAT1 inhibits autophagy and eliminates intracellular ROS mainly by binding to FUS. FUS is an RNA-binding protein, which can promote the production of hnRNA in cells, thereby promoting the transcription of related genes [30]. In this study, we showed that FUS can promote the transcription of *LC3B*. LC3B is a key protein involved in autophagy. It mainly exists on the membrane of autophagosomes. When LC3A changes to LC3B, autophagosomes are formed. Furthermore, P62 binds to LC3B and autophagosomes on the membrane of a corpuscle; as the autophagosome enters the lysosome, it is degraded [31–33]. We believe that autophagy exerts cytotoxicity in prostate cancer. The results of the present study show that after binding to FUS, ACAT1 did not change FUS expression but inhibited FUS from entering the nucleus, thereby inhibiting the production of the autophagy-specific protein LC3B. We speculate that P62 accumulation might be caused by the damage that occurs in the later stages of autophagy [34], which prevents P62 from entering the autophagolysosome, and thereafter, its degradation. Nrf2 is located downstream of P62 [35, 36]. Nrf2 expression increased with the increase in P62 expression, and the active oxygen was scavenged.

## Conclusions

Although the mechanism by which ACAT1 promotes prostate cancer has been demonstrated, the mechanism by which ACAT1 prevents FUS from entering the nucleus needs to be further explored. Furthermore, the underlying mechanism of how FUS affects *LC3B* transcription has not been elucidated and requires further investigation. Nonetheless, we demonstrated that the ACAT1-FUS complex plays a crucial role in prostate cancer development, which provides insights into the development of new therapeutic strategies by targeting or inhibiting the formation of this complex in prostate cancer.

## Supplementary Information

**Additional file 1: Fig. S1.** *ACAT1* is an oncogene in prostate cancer. A. Edu assay showing changes in the proliferation abilities of LNCaP and PC3 cells after an increase in ACAT1 expression. *P < 0.05, **P < 0.01. B. Transwell cell migration assay showing changes in the migration ability of LNCaP and PC3 cells after an increase in FUS expression. *P < 0.05, **P < 0.01.

**Additional file 2: Fig. S2.** Associated expression of ACAT1 in prostate cancer cells. A. Expression of ACAT1 and FUS in four common prostate cancer cell lines (LNCaP, PC3, DU145, and 22RV1). The statistical analysis of gray values is shown. B. Expression of ACAT1 in the nucleus and cytoplasm. The statistical analysis of gray values is shown. *P < 0.05, **P < 0.01. C. In q-PCR assay, the mRNA levels of Nrf2 and P62 did not change significantly after an increase in ACAT1 expression in LNCaP cells and PC3 cells. D. In q-PCR assay, ACAT1 and FUS expression was increased in LNCaP and PC3 cells at the same time, whereas the mRNA levels of Nrf2 and P62 did not change significantly. E. Changes in autophagy-related protein expression after a decrease in ACAT1 expression. The statistical analysis of gray values is shown. *P < 0.05, **P < 0.01.

**Additional file 3: Fig. S3.** Changes in the FUS protein level when ACAT1 and FUS expression is elevated simultaneously. A. Western blot showing changes in the FUS protein level in LNCaP cells when ACAT1 and FUS expression was elevated simultaneously. B. Western blot showing changes in the FUS protein level in PC3 cells when ACAT1 and FUS expression was elevated simultaneously.

## Data Availability

The datasets used and/or analyzed during the current study are available from the corresponding author on reasonable request.

## Abbreviations

ACAT1: Acetyl-CoA acetyltransferase 1
ALS: Amyotrophic lateral sclerosis
FUS: Fused in sarcoma
GEPIA: Gene expression profiling interactive analysis
ROS: Reactive oxygen species
SPF: Specific pathogen-free
