# Supplementary figures and images for: Autophagy inhibition and reactive oxygen species elimination by acetyl-CoA acetyltransferase 1 through fused in sarcoma protein to promote prostate cancer
Source: BMC Cancer. 2022 Dec 14;22:1313. doi: 10.1186/s12885-022-10426-5 (PMC9753422; doi:10.1186/s12885-022-10426-5)

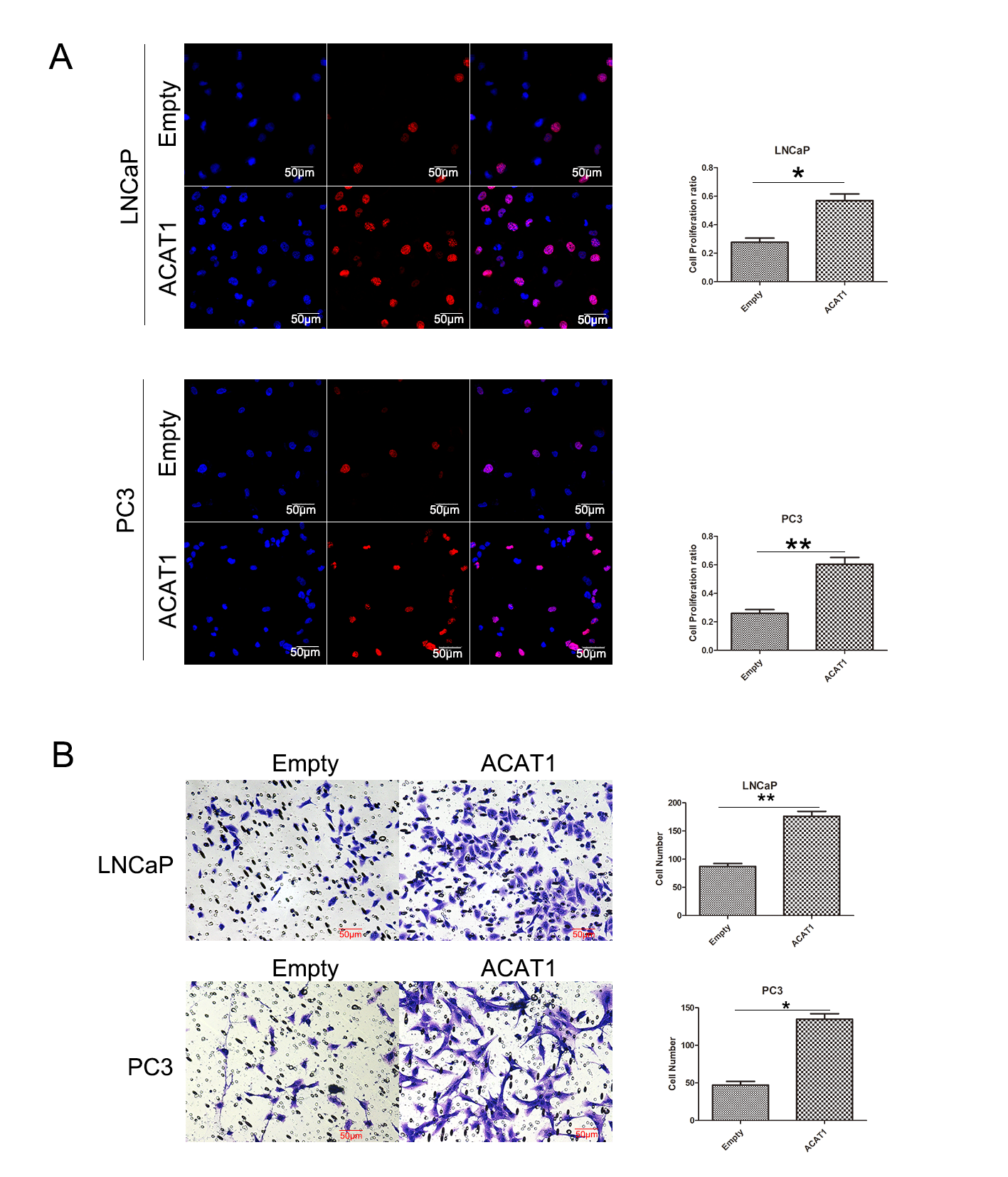

Supplement: Supplementary file 1 — Additional file 1: Fig. S1. ACAT1 is an oncogene in prostate cancer. A. Edu assay showing changes in the proliferation abilities of LNCaP and PC3 cells after an increase in ACAT1 expression. *P < 0.05, **P < 0.01. B. Transwell cell migration assay showing changes in the migration ability of LNCaP and PC3 cells after an increase in FUS expression. *P < 0.05, **P < 0.01. [file 12885_2022_10426_MOESM1_ESM.tif]

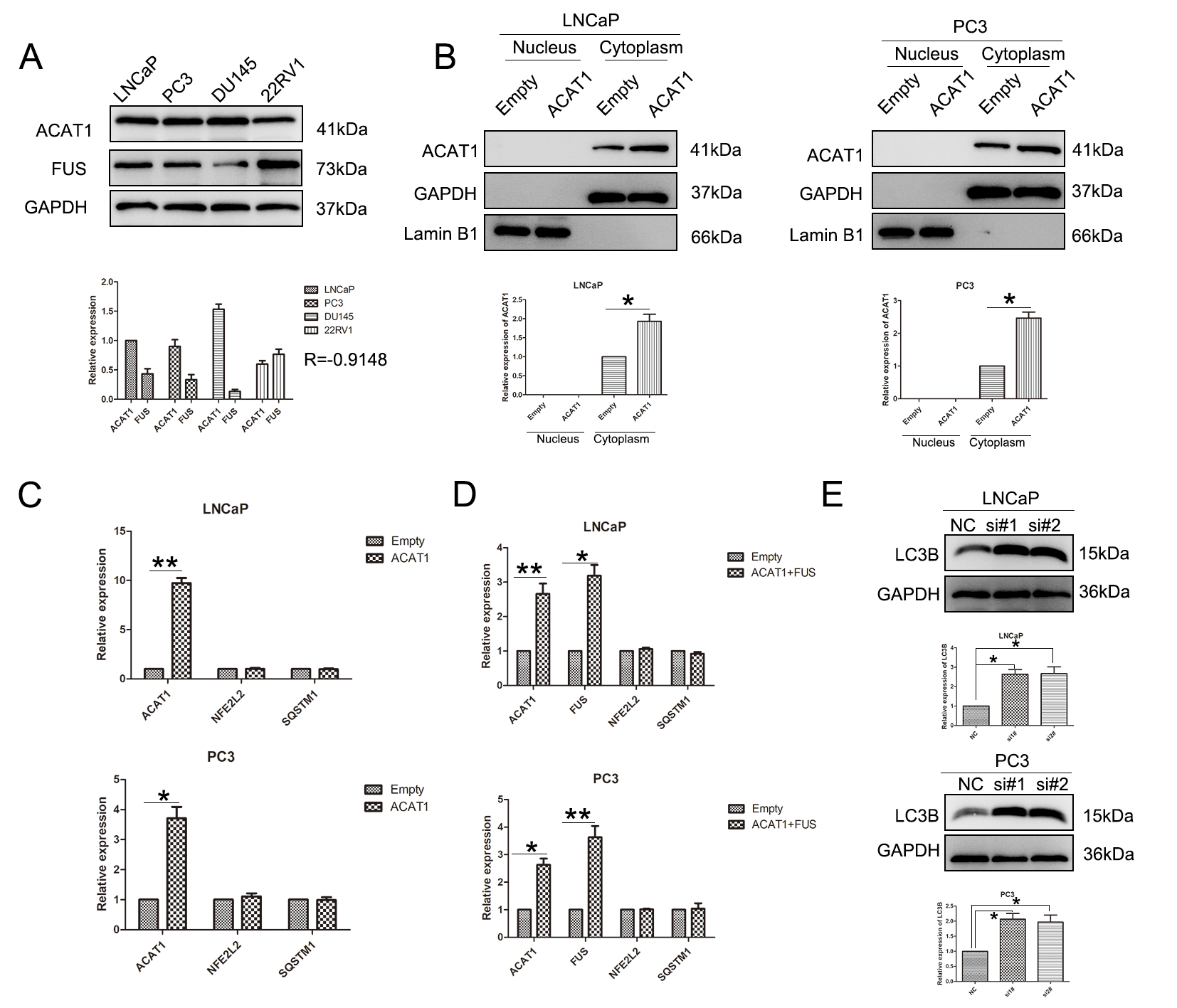

Supplement: Supplementary file 2 — Additional file 2: Fig. S2. Associated expression of ACAT1 in prostate cancer cells. A. Expression of ACAT1 and FUS in four common prostate cancer cell lines (LNCaP, PC3, DU145, and 22RV1). The statistical analysis of gray values is shown. B. Expression of ACAT1 in the nucleus and cytoplasm. The statistical analysis of gray values is shown. *P < 0.05, **P < 0.01. C. In q-PCR assay, the mRNA levels of Nrf2 and P62 did not change significantly after an increase in ACAT1 expression in LNCaP cells and PC3 cells. D. In q-PCR assay, ACAT1 and FUS expression was increased in LNCaP and PC3 cells at the same time, whereas the mRNA levels of Nrf2 and P62 did not change significantly. E. Changes in autophagy-related protein expression after a decrease in ACAT1 expression. The statistical analysis of gray values is shown. *P < 0.05, **P < 0.01. [file 12885_2022_10426_MOESM2_ESM.tif]

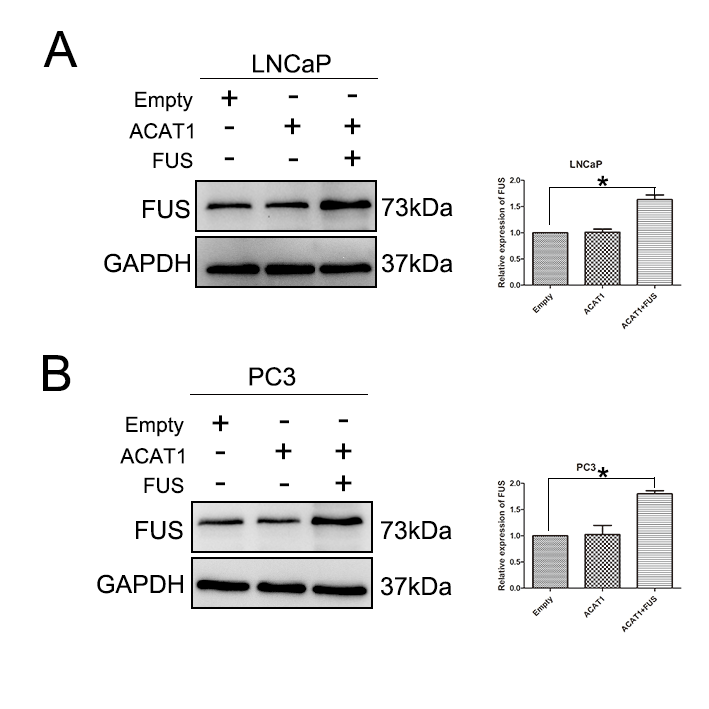

Supplement: Supplementary file 3 — Additional file 3: Fig. S3. Changes in the FUS protein level when ACAT1 and FUS expression is elevated simultaneously. A. Western blot showing changes in the FUS protein level in LNCaP cells when ACAT1 and FUS expression was elevated simultaneously. B. Western blot showing changes in the FUS protein level in PC3 cells when ACAT1 and FUS expression was elevated simultaneously. [file 12885_2022_10426_MOESM3_ESM.tif]
